# Supplementary material for: Are carbon emissions trading and green financial instruments synergistic? -Comprehensive quantitative research based on content analysis
Source: PLoS One. 2024 Mar 7;19(3):e0298601. doi: 10.1371/journal.pone.0298601 (PMC10919601; doi:10.1371/journal.pone.0298601)
Supplement: S1 Data — (ZIP) [file pone.0298601.s001.zip › Data/地级市Z_F工作报告.zip!/┴╔─■/╡ñ╢1⁄2╩╨/└┤╫╘╣1⁄2╓┌║┼í╛┬φ┐╦╩2╛▌═°í┐_2018.docx]

各位代表：

　　现在，我代表市政府向大会报告工作，请予审议，并请市政协委员和各位列席人员提出意见和建议。

　　一、本届政府工作回顾及2017年重点工作

　　五年来，在市委的正确领导下，市政府团结带领全市广大干部群众，深入学习贯彻习近平新时代中国特色社会主义思想，统筹推进“五位一体”[1]总体布局，协调推进“四个全面” [2]战略布局，持之以恒落实新发展理念和“四个着力”[3] “三个推进” [4]，在聚焦振兴中奋力拼搏，在抢抓机遇中乘势而上，在转型升级中砥砺前行，在全面从严治党中凝心聚力，各项工作不断取得新成绩，为全面建成小康社会奠定了坚实基础。

　　过去的五年，我们始终坚持以稳中求进为总基调，经济发展进入新阶段。面对经济增长速度换挡期、结构调整阵痛期和前期刺激政策消化期“三期”叠加，我们坚持发展不动摇，坚持做实经济数据，坚持在经济新常态下奋力前行。地区生产总值稳定在全省第9位;预计累计完成规模以上工业增加值929亿元，固定资产投资3036亿元，社会消费品零售总额2524亿元，外贸出口总额910亿元，一般公共预算收入469亿元。经济实力进一步增强，县域经济发展水平居全省前列，城镇化率达到66%。沈丹客运专线、丹大快速铁路、丹东机场新航站楼、丹东港20万吨级矿石泊位等一批对丹东经济社会和人民群众生活产生重大影响的基础设施项目建成并投入使用，为丹东全面振兴发展提供了强有力的基础保障。

　　过去的五年，我们始终坚持以创新驱动为引领，产业结构呈现新格局。现代农业稳步推进，粮食产量连续四年突破百万吨大关，设施蔬菜产量稳定在50万吨;特色农业蓬勃发展，农产品出口居全省前列，形成了特色鲜明的农业产业体系。工业转型升级步伐加快，建成省内首个中科院产业技术创新与育成中心;高新技术企业达到91户，高新技术增加值年均增长10%以上，居全省前列;东方测控、克隆集团、东发集团等一批“小巨人”[5]和“专精特新”[6]企业不断做大做强;获得“中国满药之都”“中国(凤城)工业内燃机增压器产业生产基地”“中国(东港)运动户外服装名城”3个国家级区域产业品牌;拥有中国驰名商标10件、国家地理标志商标10件。现代服务业发展势头强劲，建成省级服务业集聚区5个、夜经济集聚区9个，以及万达广场、华美新天地等一批城市综合体;全市电子商务交易额年均增长30%以上，东港成为国家电子商务进农村示范县;金融业增加值年均增长8%以上，引进金融机构22家，在“新三板”挂牌企业6家;预计全市旅游收入年均增长14.7%，宗裕温泉旅游度假区、凤上线旅游专列等大项目陆续投入使用，凤凰山景区旅游综合收入连续两年超亿元，旅游业总体发展水平居全省前列。

　　过去的五年，我们始终坚持以合作共赢为原则，对外开放开创新局面。辽宁沿海经济带“黄海翼”发展活力进一步增强，沿海区域形成了经济带、产业带和旅游带，重点园区规划面积扩大到194.5平方公里，以丹东新区为龙头的新兴开发区域实现项目建设和功能完善同步推进。对外贸易结构不断优化，引进外资质量持续提升，曙光汽车、时代龙增压器等企业在美国、加拿大、阿联酋的海外营销网络投入使用。与东北东部城市群区域合作日益加强，被海关总署批准为全国首批出境加工试点地区，国门湾中朝边民互市贸易区投入运营。丹东港现有泊位49个，年吞吐能力达到1.6亿吨。

　　过去的五年，我们始终坚持以深化改革为统领，经济社会发展焕发新活力。积极推进政府职能转变，持续深化“放管服” [7]改革，清理行政职权869项，审批效率提高了30%，实现“双随机、一公开”[8]全覆盖。农村土地确权、多证合一、“个转企”、出口联检“三互”[9]等改革走在全省前列，财税、商事制度、医疗卫生、文化教育、国有企业、机关事业单位养老保险等领域改革稳步推进，共完成改革任务400多项，主要领域改革主体框架基本确立。

　　过去的五年，我们始终坚持以绿色发展为遵循，生态建设取得新成效。持续开展“四城联创”工作，大力实施碧水、蓝天、青山、沃土工程，在全省率先实施乡镇环境卫生综合整治，开展了力度空前的取缔黄标车、拆除燃煤小锅炉、秸秆禁烧、治理城市内河和“两退一围”[10]、耕地培肥等环保行动。丹东成为中国十佳空气品质城市、全国水生态文明建设试点城市，生态环境居全省前列，用实际行动践行了“绿水青山就是金山银山”的理念。

　　过去的五年，我们始终坚持以增进福祉为目的，人民生活发生新变化。预计城镇和农村常住居民人均可支配收入年均分别增长8%和9.1%。民生支出比重保持在75%以上。脱贫攻坚战取得决定性进展，实现17.7万人脱贫、84个贫困村“摘帽”。改造棚户区177万平方米、老旧小区109个、农村危房2.2万户。全面推进大众创业、万众创新，实名制就业累计达到25.9万人。实现养老金“十三连涨”、城乡低保“十二连涨”，基本建成覆盖城乡居民的社会保障体系。开展了大范围的供水供气供热排水管网改造。完成了城区铁路公路平交“卡脖”道口拓宽、城市出入口改造、四号干线锦江山隧道和六道沟转盘双向贯通等工程。教育、文化、体育、卫生、食品药品安全等各项社会事业得到全面加强。兴边富民行动和少数民族发展百乡万户惠民工程深入实施。连续七轮荣获“全国双拥模范城”称号。群众性精神文明创建活动扎实开展。社会治理体系更加完善。

　　过去的五年，我们始终坚持以人民满意为追求，政府建设展现新风貌。认真开展党的群众路线教育实践活动和“三严三实”专题教育，推进“两学一做”学习教育常态化制度化，坚决贯彻上级决策部署。自觉接受市人大及其常委会的依法监督和市政协的民主监督，主动听取民主党派、工商联、无党派人士和人民团体的意见建议，共办理人大代表建议1319件、政协提案1648件，办结率、满意(基本满意)率均达到100%。法治政府建设进一步加快，认真开展地方政府立法工作，全面清理规范性文件;依法规范行政权力运行，政务公开不断向基层延伸。行政监察和审计监督有效加强，党纪政纪处分3243人次。“七五普法”全面启动。医患纠纷等人民调解工作成效显著。服务政府、责任政府、效能政府、诚信政府、廉洁政府建设全面推进。

　　2017年，是丹东经济社会发展中极不平凡的一年。在市委的正确领导下，我们经受住了半岛形势复杂多变、经济形势起伏不定、养老金支付困难、重点企业经营风险等重大考验和严峻挑战，确保了全市经济平稳运行、社会安全稳定。预计全市完成地区生产总值800亿元，同比增长4.5%;规模以上工业增加值89亿元，增长7%;固定资产投资256亿元，下降8.6%;社会消费品零售总额571.8亿元，增长5.5%;外贸出口总额156亿元，占全省比重5.1%;一般公共预算收入71.9亿元，增长5%;城镇常住居民人均可支配收入27800元，增长6.5%;农村常住居民人均可支配收入14390元，增长7%。具体表现为：

　　(一)三次产业协调发展，经济质量稳步提升

　　农业进一步做精。划定270万亩永久基本农田。调减玉米种植面积5.7万亩，新增设施种植业面积3266亩，规模以上农产品加工企业达到148家，农产品精深加工比重达到30%。贝类、大米、草莓、燕红桃等特色农产品走上品牌发展之路，东港草莓跻身全国区域品牌价值百强，“东港大黄蚬”列入第一批中国/欧盟互认地理标志保护清单。预计农产品出口创汇达到7亿美元。

　　工业进一步做强。深入实施创新驱动战略，装备制造业走向高端化、智能化，初步形成国家、省、市三级智能制造试点示范体系。思凯电子成为国内行业物联网产品占比最高的龙头企业;曙光集团成为国家两化融合[11]管理体系贯标试点企业，启动与华泰汽车公司重组;黄海新能源汽车型号超过30个，产量超过千辆。40余家企业与20余家科研院所精准对接，签约合作项目20余个;举办大众创业、万众创新系列活动，全市众创空间达到12家。培育“小巨人”和“专精特新”企业76户，规模以上工业企业达到336户。凤城现代产业园区升级为省级经济开发区。孔雀表业、欣时代生物医药荣获“中国轻工业百强企业”称号，丹东药业成为全省唯一获得省长质量奖的制药企业，全市27项产品成为辽宁名牌产品，轻工业规模由全省第9位上升到第5位，轻工业振兴初见成效。

　　服务业进一步做优。推进全域旅游，爱河生态公园建设、蒲石河森林公园升级改造等重点旅游项目实现开工，北黄海温泉度假村被评为国家五星级温泉度假村，大鹿岛、獐岛升级为“国家4A级景区”，《丹东边境旅游试验区建设实施方案》报国务院待批，我市成为“旅游厕所革命先进市”。培植新业态新模式，推动传统商贸服务业向高端化、高附加值、高带动性方向发展。现代物流快速发展，东大货运等一批临港第三方物流企业规模逐步扩大。电子商务交易额突破200亿元，东港草莓80%实现了网上交易。实现各类融资230亿元，引进招商证券等金融机构6家。万达金街、安东老街、女人街成为夜经济亮点。

　　(二)招商引资项目建设年活动深入开展，有效投资持续发力

　　招商引资富有成效。围绕港口建设、高效农业、城市基础设施等方面，科学谋划、包装、推介项目300余个。开展市级“走出去，请进来”招商活动70余次，与恒基伟业、中高集团、东软集团、锦江集团、大欧化成株式会社等企业深入合作，签约落地大项目80余个。预计引进内资到位资金240亿元，实际利用外资1.5亿美元。

　　项目建设加快推进。采取层层签订责任状、包扶重点项目、现场拉练、调度通报等措施，积极推进项目建设。全市5000万元以上开复工项目233个，计划总投资1285亿元，其中：亿元以上开复工项目201个，计划总投资1264亿元;省政府重点推进的13个项目全部实现开复工。总投资7.8亿元的王老吉夏桑菊饮料、总投资4.7亿元的廉家坝光伏发电(一期)、总投资4.2亿元的金山热电固体电蓄热调峰等一批项目实现竣工;总投资5.6亿元的鸿润养老机器人、总投资5亿元的康辰药业产业园、总投资5亿元的中地牧业生态牧场等一批项目开工建设。

　　营商环境持续优化。制定出台《关于进一步加强优化营商环境建设的意见》《营商环境企业举报保护制度》《打造企业公平政策环境专项工作方案》，以问题为导向，开展涉企收费、政府机构失信、涉金融企业失信和干部作风等问题专项整治活动，处理涉及营商环境案件109起。整合成立不动产登记和房屋交易大厅，新建的市民行政服务中心投入使用，硬件设施达到省内一流水平，初步实现“三集中、三到位”[12]。推进政务审批智能化，启动工商登记全程电子化改革，实现“26证合一、一照一码”，完成“个转企”739户。营商环境优化使4万余户企业和商家受益。

　　(三)各项改革全面实施，发展动能更加强劲

　　扎实推进供给侧结构性改革。出台三次产业等5个重点领域的供给侧结构性改革实施意见。坚决落实“三去一降一补” [13]任务，在全省率先关闭9万吨以下小煤矿42个，化解煤炭产能185万吨;拆除落后炼铁小高炉1座，化解生铁产能27万吨;淘汰燃煤锅炉288台、黄标车及老旧车辆7974台;化解房地产库存面积231万平方米。全面落实减税降费政策，共为企业减轻负担18.2亿元。加快补齐民生和社会事业短板。

　　稳步推进重点领域改革。实施城市管理执法体制改革;完成交通、文旅、城建3家国有企业集团组建，12家国有企业公司制改造，18家市本级生产经营类事业单位改革，8家驻丹央企“三供一业”主辅分离移交[14]和13个国有林场改革。农村土地承包经营权确权率达到96%。其他领域改革正在全面推进。

　　(四)对外开放进一步扩大，区域合作日益活跃

　　沿海经济稳步发展。重点开发区和园区建设加快推进。合作区、高新区、大孤山经济区管理体制得到调整优化，机构编制大幅削减，突出了招商引资和项目建设职能。丹东高新区创建国家高新区已上报国务院待批。丹东综合保税区申报按国家“一区一园”新要求调整选址。临港产业加快发展，黄海汽车、本钢不锈钢、哈曼科技等重点企业主营业务收入均实现了两位数增长。

　　区域合作步伐加快。全市25家企业参与“一带一路”沿线国家投资。深入对接吉林向南发展战略[15]，与东北东部地区在经贸、旅游、物流等方面的交流合作日益增多，《东北东部绿色经济带发展规划》近期有望获得国务院批准。丹东港通化国际内陆港务区实现通关运营。携手通化、白山成立鸭绿江旅游联盟，共同打造鸭绿江国际旅游品牌。启动与扬州市对口合作，签订了城市旅游合作框架协议。积极开展对口援疆工作。

　　外贸出口总体稳定。全力向上争取政策和资金，支持企业开拓日韩、东南亚、欧美等市场，组织企业参加国内外展销会，扩大订单，对日本、韩国、东盟、欧盟等贸易伙伴国的出口均保持两位数增长，打开了外贸出口新局面。

　　(五)城市功能逐步完善，城乡环境质量明显提高

　　基础设施持续改善。三湾水利实现向城区供水，结束了丹东建成区单一水源的历史。启动新区污水处理厂建设和城市黑臭水体综合整治工程。疏港高速公路、丹大高速前阳出口竣工通车，黄海大道、十纬路、铁矿路、兴五路等40余条城市道路完成改造提升，站前地下通道竣工。市区集中供热联网面积达到3680万平方米。新增新老城区快速公交线路3条，全市绿色环保公交车辆占比达到83%。实施公园绿地、城市面山、主要街路绿化提升工程，完成锦江山至元宝山景观绿道(一期)和青年湖公园改造。

　　城市管理持续加强。启动城市风格总体规划纲要编制，加大市容环境卫生综合整治力度，拆除清理各类违规广告牌匾5.5万块(处)，清理店外堆占、占道经营4.5万处，拆除违章建筑1000余处。完成大沙河河道治理和黑沟水库水源入劈柴河工程。城市污水集中处理率达到72.6%，生活垃圾无害化处理率达到95%。国家卫生城市创建通过“国检”。继续保持全国文明城提名城市。

　　人居环境持续优化。全面落实中央环保督察和国家海洋督察整改任务，加快推进大气、水、土壤等环境整治，空气质量优良天数居全省首位，河长制全面推行，水源地水质达标率达到100%，《丹东鸭绿江口湿地国家级自然保护区管理条例》成为我市首部实体性地方法规。特色乡镇建设初见成效，大梨树、三道湾被评为国家级“美丽宜居村庄”，大鹿岛被评为“全国特色民俗村”，獐岛被评为“全国最美渔村”。

　　(六)民生工作得到加强，社会事业全面进步

　　社会保障能力显著提升。城镇新增就业1.9万人，城镇登记失业率控制在4.2%以内。全市11万建档立卡贫困人口、低保对象、特困人口等困难群体全部纳入社会保险保障范围，符合条件的人员可以直接领取城乡居民养老保险基础养老金。新农合基本实现全覆盖。完成棚户区住房改造4642套、农村危房改造2140户。发放残疾人“两项补贴”3100余万元。

　　脱贫攻坚扎实推进。认真落实“六个精准”[16]“五个一批” [17]要求，投入财政资金近6000万元，通过发展产业、促进就业、加强教育、改善住房、低保兜底等措施，实现1.5万人脱贫、42个贫困村“摘帽”。

　　社会事业蓬勃发展。启动13家城市公立医院综合改革并全部取消药品加成;12家医保定点医疗机构与全国7443家医院实现联网直接结算;新增54家社会办医疗机构。32所乡镇中心幼儿园实现提质晋级;33所农村义务教育薄弱学校改造完成;丹东市示范性综合实践基地、丹东七中、六纬路小学、金汤小学、经山小学、实验小学新区分校、第四幼儿园等新建、改扩建工程竣工投入使用;丹东职专通过国家级示范校验收。组织“流动图书馆”“流动大舞台”等文化服务活动137场次。全民健身活动广泛开展，健身器材、笼式运动场走进乡村。加强食品安全监管，560家持证学校食堂“明厨亮灶”改造率达到100%。

　　(七)风险防控措施得力，边境地区安全稳定

　　财政金融风险有效管控。积极拓展财力来源，一般性支出压减5%，“三公”经费实现只减不增，集中财力“保工资、保运转、保民生”。化解存量政府性债务15.8亿元;当年置换债券50亿元，节约利息2亿元。完成互联网金融风险专项整治。全力帮助重点企业化解债务风险。

　　边境地区安定和谐。不断完善党政军警民“五位一体”管边控海新机制，严厉打击走私和非法易物活动，开展了空前严厉的伏季休渔管理、制止非法到敏感水域捕捞和涉渔“三无”船舶整治行动，拆解取缔涉渔“三无”船舶420艘。实施了“春雷”“畅安”等严打专项行动，持续开展安全生产大检查和公共安全大整治活动，保持了社会安全稳定。积极稳妥应对H7N9流感疫情，未发生农畜产品质量事故。应急保障能力进一步提升，科学周密应对“8·4”强降雨，提前转移安置群众1.62万人，确保了人民群众生命安全。扎实开展信访矛盾“清仓起底”化解攻坚行动，实现了“减存控增”目标。国防动员、国防教育和民兵预备役工作取得新成绩。

　　外事、侨务、台务、接待、老龄、档案、气象、公积金、地方志、防震减灾等工作也取得了新的进展。

　　各位代表，五年来全市经济社会取得的成绩，是市委总揽全局、正确领导的结果，是市人大、市政协有效监督、全力支持的结果，是全市广大干部群众共同努力、不懈奋斗的结果。在此，我代表市政府，向辛勤工作在各个领域的同志们，向人大代表、政协委员，向各民主党派、工商联、无党派人士和各人民团体，向中省直部门、解放军驻丹部队和武警官兵，表示衷心的感谢并致以崇高的敬意!

　　在肯定成绩的同时，我们也清醒地看到当前全市经济社会发展中还存在一些问题。主要表现为：一是经济下行压力依然较大，固定资产投资和外贸出口增长乏力;二是经济发展质量和效益还不高，创新能力不强，新旧动能转换不快;三是养老金支出、政府债务负担较重，中小企业融资难问题依然存在;四是发展不平衡、不充分的一些突出问题尚未有效解决，城乡居民收入偏低，民生领域仍有短板;五是营商环境有待进一步优化，少数干部担当精神不足。这些问题我们将在以后的工作中，采取更加有力的措施认真加以解决，让全市人民感受到实实在在的变化和成效。

　　二、新一届政府工作的总体思路和奋斗目标

　　各位代表，未来五年是我们深入学习贯彻党的十九大精神、“十三五”规划顺利实施、决胜全面建成小康社会的重要时间节点，也是“两个一百年”奋斗目标的历史交汇期。新时代新起点需要新作为，我们将坚决落实市委第十二次党代会精神，紧紧抓住新一轮东北振兴、辽宁沿海经济带建设、沿边地区开发开放、东北东部区域协同发展的重大叠加机遇，推动经济转向高质量发展。今后五年政府工作的总体思路是：深入贯彻落实习近平新时代中国特色社会主义思想和党的十九大精神，统筹推进“五位一体”总体布局，协调推进“四个全面”战略布局，自觉践行新发展理念，按照“四个着力”“三个推进”的要求，大力发展实体经济，持续深化供给侧结构性改革，加快辽宁沿海经济带“黄海翼”建设和沿边地区开发开放，促进城乡融合，增进民生福祉，努力实现更高质量、更有效率、更加公平、更可持续的发展，与全国同步建成小康社会，加快丹东新一轮全面振兴发展。

　　根据上述总体思路，今后五年政府工作的主要奋斗目标是：

　　——经济实力实现大幅提升。坚持经济发展质量和效益“双提升”，增速不低于全省平均水平。现代化经济体系基本构建，乡村振兴战略全面实施，县域经济比重明显提升;重振轻工业名城雄风，跻身全省品牌大市行列;传统服务业升级改造成效显著，现代服务业实现做大做强。本宽高速、沈丹铁路电气化改造等一批投资10亿元以上的重大项目开工建设。“十三五”规划顺利实施，到2020年全面建成小康社会，开启社会主义现代化建设新征程。

　　——改革开放谱写新篇章。创新驱动发展战略深入实施。各领域改革取得明显成效。营商环境走在全省前列。国家高新区获批，综合保税区建设初见成效，丹东重点开发开放试验区开始建设，充分发挥港口在开放体系中的优势，主动全面融入“一带一路”、辽宁沿海经济带、辽宁自贸试验区、东北东部经济带发展，努力把丹东打造成为富有特色的现代化国际港口城市。

　　——新老城区共同繁荣。加快推进“多规合一”[18]。城市服务功能明显增强，爱河水更大范围进入千家万户，天然气等清洁能源得到广泛应用，城市集中连片棚户区彻底消灭，城市老旧险桥改造完成，四号干线主城区段全线通车，客运站点和公交线路布局优化，公交车和出租车全部更换为清洁能源汽车。加快新老城区连接带建设，适时启动鸭绿江新老城区段水上客运交通工程。新区城市功能基本完善，交通便利，商业繁荣，国际会展城、新区医院、新区中学等投入使用，实现新老城区功能互补，错位发展，努力使新区成为丹东行政经济中心。

　　——生态环境争创全国一流。坚持绿色发展，实施最严格的生态环境保护制度，生态环境质量持续良好，森林覆盖率达到66%，空气质量优良天数率达到85%，城市污水集中处理率达到95%，城市内河黑臭水体整治和单位GDP能耗、二氧化碳排放降低任务全面完成，生态安全屏障基本形成，打造省内乃至国内一流的生态文明城市。

　　——人民群众安居乐业。城乡居民收入增长与经济增长同步，常住人口城镇化率达到68%，全面建成覆盖全民、城乡统筹、权责清晰、保护适度、可持续的多层次社会保障体系。教育、文化、体育、卫生等社会事业全面发展。完成食品安全城市创建。积极争创第八轮“全国双拥模范城”。坚决打赢脱贫攻坚战，完成农村危房改造任务。新型智慧城市框架基本建成。社会主义核心价值观融入社会发展各方面。形成有效的社会治理、良好的社会秩序，使人民获得感、幸福感、安全感更加充实、更有保障、更可持续。

　　为了实现上述目标，新一届政府在工作中将切实做到“八个坚持”：一是坚持把学习贯彻党的十九大精神作为全市首要政治任务，让上级决策部署在丹东落地生根。二是坚持把建成全面、高水平、百姓认可的小康社会作为本届政府头等大事，让改革发展成果更多更公平惠及人民群众。三是坚持把新发展理念作为丹东全面振兴发展的科学路径，促进经济高质量增长;四是坚持把供给侧结构性改革作为丹东今后一个时期经济社会的发展主线，进一步激发内生动力。五是坚持把对外开放作为丹东全面振兴发展的必由之路，持续提升开放型经济发展水平。六是坚持把生态文明建设作为美丽丹东建设的根本遵循，使生态优势尽快转化为经济优势。七是坚持把精细化管理作为提升城市品位的重要抓手，不断增强城市规划建设管理能力。八是坚持把维护边境安全稳定作为丹东发展改革的前提保障，全力做好边海防各项工作。

　　三、2018年全市经济社会发展目标任务和重点工作

　　各位代表，今年是学习贯彻党的十九大精神及新一届政府履职的开局之年，是决胜全面建成小康社会、实施“十三五”规划承上启下的关键一年。我们将以市委十二届八次全会精神为指导，坚持稳中求进工作总基调，统筹推进稳增长、促改革、调结构、惠民生、防风险各项工作，推动质量变革、效率变革、动力变革，在打好防范化解重大风险、精准脱贫、污染防治攻坚战方面取得扎实进展，促进经济社会持续健康发展。

　　今年经济社会发展的主要预期目标是：地区生产总值同比增长6.5%，规模以上工业增加值增长7%，固定资产投资增长10%，社会消费品零售总额增长7%，外贸出口总额占全省比重4%，一般公共预算收入增长5%，城镇常住居民人均可支配收入增长7%，农村常住居民人均可支配收入增长7%。

　　为实现上述目标任务，我们将全力抓好以下重点工作。

　　(一)坚持创新驱动发展，大力振兴实体经济

　　加快发展更有特色、更加高效的现代农业。推动粮经饲三元种植结构协调发展，调减玉米种植面积1万亩，优化畜牧业布局，壮大林下经济，扩大小浆果、中药材等特色产业规模，做大水产品、果蔬等农产品加工产业集聚区，农作物耕种综合机械化水平达到74%。实施育繁推一体化，加快推进丹玉种业、辽丹种业上市。推动海洋产业转型升级，支持远洋捕捞业发展和健康生态养殖，建设“海上粮仓”。优化提升6个省级以上农产品出口示范区，农产品出口额稳定在7亿美元。

　　加快发展更高质量、更具竞争力的先进制造业。实施创新驱动发展战略，大力发展战略性新兴产业，全面落实《中国制造2025丹东实施方案》，培育一批智能制造示范企业和两化融合示范企业，继续加大对中小微企业的财税、信贷支持力度，力争“小巨人”和“专精特新”企业达到110户、高新技术企业达到110户，有效发明专利拥有量达到850件。加快推进消费品产业增品种、提品质、创品牌，让“孔雀”“康齿灵”等老品牌焕发生机;打造陀飞轮、北黄海海洋生物制品、塔醋等一批新优势特色产业和产品，推动重振丹东轻工业名城雄风工作取得实质性进展。促进重点产业集群提质增效，显著提升黄海新能源汽车、曙光车桥、凤城增压器等汽车及零部件生产基地和满族医药、测控仪器等新型产业基地建设水平。

　　加快发展更有前景、更具活力的现代服务业。深入推进全域旅游，力争凤凰山玻璃索桥、爱河生态公园、宽甸朝鲜族风情温泉度假村等一批旅游项目投入运营，北黄海温泉度假村、东汤天沐君澜等养老养生项目做大做强，积极培育一批旅游特色小镇和金牌农家乐、精品民宿，包装推广丹东餐饮名店、地方名菜、特色小吃等十大品牌。切实加强景区管理，提升环境卫生质量和从业人员综合素质。促进传统流通业态转型升级，实现线上线下深度融合发展。推进特色街区建设，持续繁荣夜经济，力争夜间销售占比达到30%以上。加快发展冷链物流、城乡配送和港航服务，支持物流衍生服务发展。大力发展会展业。依托生态、旅游、养老养生、医学美容等产业，发展“美丽经济”。加大养老服务机构建设力度，加快推行社区居家养老服务。进一步改善金融环境，积极破解融资难题，实现各类融资180亿元，新建或引进金融机构2家，新增“新三板”挂牌企业1家。

　　(二)加快招商引资和项目建设，不断夯实发展基础

　　打好项目建设攻坚战。年内实施5000万元以上项目305个，计划投资总额1228亿元。其中：亿元以上项目237个，新开工项目93个。

　　——新开工基础设施项目包括总投资35亿元的沈丹铁路(丹东段)电气化改造、总投资4.5亿元的凤东线(东港段)改建、总投资2.4亿元的凤城生活垃圾焚烧发电等项目。

　　——新开工工业项目包括总投资16.8亿元的廉家坝光伏发电(二期)、总投资10亿元的沈阳机床智能制造谷、总投资10亿元的大羽有机涂层板、总投资8亿元的凤城老窖技改扩能、总投资5.1亿元的电子陶瓷封装外壳等项目。

　　——新开工服务业项目包括总投资30亿元的恒通刘家河温泉水镇、总投资12亿元的爱立信浪潮凤凰云端、总投资10亿元的抗美援朝红色文化主题公园、总投资3亿元的丹东义乌小商品城等项目。

　　——新开工农业项目包括总投资30亿元的现代渔港经济区、总投资2.6亿元的通威渔光一体现代渔业产业园等项目。

　　打好招商引资攻坚战。紧跟国家产业导向，立足资源和产业优势，通过节会招商、以商招商、产业链招商等方式，提高投资的效率和精准率。积极谋划、包装亿元以上各类项目300个。重点在总投资150亿元的锦江集团600万吨氧化铝、总投资36亿元的润恒集团冷链物流和总投资35亿元的中能建前阳燃气电站等前期招商条件成熟的大项目上实施“定向突破”。继续开展赴韩国、美国、加拿大、德国等国家招商活动。大力支持本土企业做大做强，拉长产业链条，开展二次投资和创业，实现二次腾飞。

　　打好服务保障组合拳。建立营商环境整治常态化机制，聘请第三方机构对营商环境进行调查评估，加快政府诚信、商务诚信、社会诚信和司法公信体系建设，打造高效优质的营商环境。深化园区体制机制改革，完善园区基础设施和水电气路等要素保障，招商引资项目更多向园区集聚，实现土地节约利用和上下游产业延伸发展。进一步分解、细化上级重大利好政策，有针对性地向上争取项目和资金。继续实施招商引资和项目建设目标责任制、重大项目代办制、包保责任制等，确保大多数项目在上半年开工建设。

　　(三)全面深化改革，进一步激发振兴发展动力

　　深入推进供给侧结构性改革。坚持质量强市，全面提高供给体系质量。继续去产能、去库存、去杠杆、降成本、补短板，全面落实关闭燃煤小锅炉、取缔黄标车及老旧车辆、化解房地产库存、企业减负等任务，重点在破除无效供给、培育新动能、降低实体经济成本上下功夫。坚持培养和引进相结合，实施更加积极、更加开放、更加有效的人才政策。大力弘扬劳模精神和工匠精神。

　　持续推进“放管服”改革。加强权责清单管理，推进权力清单“瘦身”和责任清单“强身”。建成“易办事”实体大厅和“e办事”网上大厅，实施联合辅导、会审、踏勘、验收的审批机制和多规多评多测合一、多图联审的服务机制，确保行政审批“三集中、三到位”，审批时限做到“3540”[19]，实现大厅之外无审批。完善“双随机、一公开”，强化事中事后监管，防止监管“缺位”和管理“真空”现象发生。

　　积极推进其他领域改革。建立全面规范透明、标准科学、约束有力的预算制度，全面实施绩效管理，切实增强财政保障能力。开通财政预算联网监督系统。新组建的国有企业集团全部实现挂牌运营。稳妥推进行政类、公益类事业单位改革。抓好土地确权登记收尾工作，全力推进农垦、供销社、国有林场、集体林权、农村集体产权、国有粮食企业等改革，继续实施投融资、教育、文化、统计、医疗卫生等领域改革，把社会领域巨大发展潜力充分释放出来。

　　(四)遵循共商共建共享原则，推动形成全面开放新格局

　　促进沿海经济提质升级。实施《丹东沿海经济带三年攻坚计划》，突出抓好各类产业园区创新发展。坚持以港兴市，依托区位、产业、资源、交通、土地等多重优势，加速优质生产要素聚集和临港产业发展，加快推进丹东港重组。依托新投产的尼龙56项目，拉长产业链条，推动5万锭纺纱配套项目建设。充分发挥对外开放平台作用，加快国门湾中朝边民互市贸易区发展，推动沿江口岸升级开放。进一步完善新区等新兴开发区域的配套设施。

　　促进区域经济合作升级。落实好《东北东部绿色经济带发展规划》，培育东北地区新的经济增长极。落实好《白通丹经济带总体发展规划》，着力加快丹通经济带建设步伐。依托辽宁自贸试验区政策红利溢出效应，积极承接沈阳、大连等地区的产业转移，在经贸发展、园区建设、旅游推广等方面不断拓宽合作空间。以旅游和园区管理为突破口，加强与扬州的对口合作。挖掘丹东与丹麦文化交流历史渊源，在文化、旅游、经贸等方面开启交流合作。

　　促进外贸出口转型升级。积极开拓日韩、欧盟、东盟和美洲市场，逐步减轻对传统市场的依赖。大力实施出口品牌战略，鼓励企业开展境外商标注册，提高出口商品的科技含量和附加值，争取东港草莓和水产品2个出口基地晋升为国家级外贸转型升级基地。

　　(五)聚力乡村振兴，加快城乡融合发展

　　坚决完成脱贫攻坚行动计划。对贫困人口实行动态精准识别，因村因户施策，推广“村集体+贫困户”“企业+贫困户”“合作社+贫困户”“种养殖大户+贫困户”等新型产业扶贫模式，确保1.2万贫困人口稳定脱贫、40个贫困村“摘帽”。

　　大力实施乡村振兴战略。促进农村一二三产业融合发展，落实《丹东市推动县域经济发展全面建成小康社会三年行动计划》《兴边富民行动“十三五”规划》，不断培育县域经济发展新动能，加快落实县域“一园一区一镇一品”，积极搭建农村电商平台，有效拓宽农民就业渠道和收入来源。加快特色乡镇和美丽乡村建设，扶持村集体经济发展，在大梨树、马市岛等地开展田园综合体[20]项目试点。大力改善水电气路房等基础设施，重点实施400公里农村公路黑色路面工程，提高农村住房抗震设防能力。持续开展垃圾、污水、畜禽粪便综合整治，农村卫生厕所普及率达到85%。加强和创新农村社会治理。促进农村文化教育、医疗卫生等事业发展。加快建设一批大梨树式的好村庄，培养一批毛丰美式的好干部。

　　持续完善城市基础设施功能。构建科学精细的城市建设与管理体系。推进生态修复和城市修补工作。巩固“四城联创”成果，加快创建国家环保模范城和全国文明城。通过增加绿地、提高硬化路面透水蓄水能力、改造老旧泵站、新建扩容泵站等措施解决城市内涝问题。实施日龙山至柳林河防洪工程。改造九纬路，延伸十纬路;提升改造滨江路、锦山大街、青年大街等城市道路和白房路、铁矿路、仁忠街等北部山区道路，维修改造桃源立交桥、胜利桥等城市桥梁，力争贯通四号干线花园路至宝山大街段，浪东线至201国道新出口建成通车，启动城市东出口和灯塔山区域改造工程。下大气力解决城市动迁遗留问题。实施鸭绿江大桥等城市重点部位亮化工程和鸭绿江景观带、锦江山公园、珍珠公园、玉带河升级改造工程。继续开展城市户外广告牌匾集中整治。完成丹东客运站维修改造，启动迎宾岭旅游车辆集散地项目建设，新建公交停车场3个以上、城市公共停车位1000个以上。实施殡仪馆升级改造工程，改善城市出入口形象。

　　(六)突出以人民为中心，让广大群众有更多的获得感和幸福感

　　让人民群众有更可靠的社会保障。实施《丹东市城乡居民和各类群体收入增长三年行动计划》。城镇新增就业1.9万人，城镇登记失业率控制在4.5%以内，“零就业家庭”保持动态为零。积极做好复转军人安置和随军家属就业等涉及官兵切身利益的工作。继续做好社会救助对象提标和残疾人就业、救助、帮扶工作。拓展社会保障卡应用范围，实施全民参保计划，进一步完善城镇职工和城乡居民基本养老保险参保缴费政策，确保养老金按时足额发放。

　　让人民群众有更舒适的居住条件。持续推进保障性安居工程，改造棚户区住房2368套、老旧小区15个、农村危房1300户。强化对物业公司的监管，提升物业服务质量。加强城乡饮用水源保护和监管，适时启动龙头山净水厂建设。

　　让人民群众有更好的教育。加大幼儿园建设和管理力度，确保普惠制幼儿园学位覆盖率达到73%。义务教育学校标准化建设达到90%，振安区和宽甸县通过国家级义务教育发展基本均衡县(市)区验收，新区高标准初中建成。2所普通高中列入省级教育教学改革典型学校。积极构建现代职业教育体系。依托“三通两平台”[21]建设，加快推进教育信息化。大力实施乡村教师支持计划，狠刹教师违规补课风。

　　让人民群众有更高水平的医疗卫生服务。协调推进“三医联动”[22]。组建4个城市医联体和7个县域内医联体。扩大新农合跨省异地就医结算覆盖面，完成60家乡镇卫生院标准化建设。加快中医药事业发展。鼓励更多的社会资本进入医疗服务领域。推行免费婚前体检。

　　让人民群众有更美的生态环境。坚持生态立市，继续保护好丹东的绿水青山蓝天，深入实施《丹东市污染防治与生态建设和保护攻坚三年行动计划》，完成鸭绿江口污染和大沙河、五道河、白房河等城市黑臭水体综合整治，建立水污染企业黄红牌公示制度，保护好地热资源。强化土壤污染管控和修复，完成农用地土壤污染详查，全市耕地保有量不低于336万亩。完成营造林20万亩。扩大高污染燃料禁燃区范围，加强秸秆禁烧和综合利用。启动领导干部自然资源资产离任审计。

　　让人民群众有更丰富的精神文化生活。立足文化自信，做足丹东文化大文章。实施文化遗产保护工程，挖掘“英雄城市文化”“边城文化”“民族文化”，在市区文物遗址设立保护标志和文化标识;抗美援朝纪念馆恢复对外开放;支持小型专业博物馆建设。实施文体惠民工程，开展基础综合性文化服务中心示范点建设，确保宽甸“辽宁最美新华书店”投入使用;继续开展全民健身活动，承办各类体育赛事，配备健身路径50套。

　　(七)全力加强风险防控，维护边境地区安全稳定

　　守住财政金融安全底线。将政府债务管理重点由置换债务转向化解债务，严格实行政府债务限额管理，全力化解存量政府性债务。继续整治非法集资、高杠杆和互联网金融风险，积极防范和化解重点企业债务风险。

　　守住安全生产底线。完善安全生产责任制，坚决防止已关闭小煤矿死灰复燃，深化非煤矿山、尾矿库、危险化学品、交通运输、建筑施工等重点领域专项整治，杜绝发生重特大安全生产事故。

　　守住边境安全稳定底线。进一步梳理和解决好信访积案，构建多元化矛盾纠纷化解机制。做好民族宗教工作。加强立体化社会治安防控体系建设，依法打击各类违法犯罪活动。加大边海防管控力度，强化“五警(军)联勤”武装巡逻，严厉打击边境走私活动，持续开展涉渔“三无”船舶整治和“江海平安”系列整治行动，坚决制止非法到敏感海域捕捞。高度重视双拥工作，推进军民融合发展。

　　四、建设人民满意的服务型政府

　　为人民服务、对人民负责、受人民监督、让人民满意，永远把人民对美好生活的向往作为奋斗目标，这是新一届政府矢志不渝的追求。我们将保持“赶考”的姿态、坚定“考好”的决心，向人民交上满意的答卷。

　　牢记使命，让十九大精神和上级决策部署在丹东落地生根。牢固树立“四个意识”[23]，坚定“四个自信”[24]，坚决维护以习近平同志为核心的党中央权威和集中统一领导，始终在思想上政治上行动上同以习近平同志为核心的党中央保持高度一致，全面落实中发7号文件[25]、国发62号文件[26]以及省“五大区域[27]发展战略”“一带五基地”[28]建设部署，坚决服从市委正确领导，让十九大精神全面贯穿于全市经济社会和政府建设中，切实把党的路线方针政策转化为推进丹东振兴发展的实际行动。

　　依法行政，将政府工作全面纳入法治和监督轨道。加强法治政府建设，依法全面履行政府职能。加强政府立法和制度建设，完善政府法律顾问制度。落实行政行为监督机制，高度重视并认真办理人大代表建议和政协提案。严格规范公正文明执法，强化执法监督。提高政务公开制度化、标准化水平，让权力在阳光下运行。

　　真抓实干，使大梨树“干”字精神在丹东发扬光大。大梨树的“干”字精神是从丹东走向全国的，也是在政府工作中必须大力践行并发扬光大的。我们将以“实”字为先，“干”字当头，勇于担当，积极作为，机遇面前不迟疑，矛盾面前不回避，困难面前不退缩，以“马上就办”的作风，抓一件是一件，干一件成一桩。切实增强政府公信力和执行力，提高为发展服务、为基层服务、为群众服务的意识和本领。

　　久久为功，作风建设永远在路上。坚持全面从严治党，严格执行中央八项规定精神，坚决纠正“四风”新表现。坚持“三严三实”、民主集中制原则和“三会一课”制度，开展“不忘初心、牢记使命”主题教育。用好督查的“利器”和考核的“指挥棒”，消除工作落实的堵点和盲区。健全工作激励机制，增强干部干事创业本领，鼓励创新，宽容失败，让广大干部想干事、敢干事、干成事。加强审计监督工作。持续开展政府系统作风整治，坚决向慵懒散、不作为、慢作为、不诚信等问题“亮剑”，树立为民务实清廉的良好政府形象。

　　各位代表，实干成就梦想，奋斗赢得未来。丹东已站在新的历史起点上，让我们高举中国特色社会主义伟大旗帜，以习近平新时代中国特色社会主义思想为引领，坚定信心、奋发有为，不忘初心、继续前进，为决胜全面建成小康社会、实现丹东新一轮全面振兴发展而努力奋斗!
